# How Tourists’ Perception Affects Travel Intention: Mechanism Pathways and Boundary Conditions

**DOI:** 10.3389/fpsyg.2022.821364

**Published:** 2022-06-16

**Authors:** Xiufang Jiang, Jianxiong Qin, Jianguo Gao, Mollie G. Gossage

**Affiliations:** ^1^College of Economics, Southwest Minzu University, Chengdu, China; ^2^College of Historical Culture & Tourism, Southwest Minzu University, Chengdu, China; ^3^College of Earth Sciences, Chengdu University of Technology, Chengdu, China; ^4^Department of Anthropology, University of Wisconsin-Madison, Madison, AL, United States

**Keywords:** psychology, tourists’ perception, tourism, travel intention, integrated model

## Abstract

Tourist subjectivities have an important effect on behavioral intentions. Under the background of normalization, tourism decision-making manifests primarily in tourists’ individual preferences, which has led much research to ignore the importance of other subjective factors, as well as objective environmental factors. In the COVID-19 era, tourism behavior’s social attributes have become more prominent; the effect of important others or organizations’ attitudes toward tourism behavior, as well as personal knowledge, ability, and experience in preventing and controlling tourism risks, are evident. This study integrates knowledge-attitude-behavior (KAB), Theory of Perceived Risk (TPR), Social Identity Theory (SIT), and Theory of Planned Behavior (TPB), along with a comprehensive framework method, to construct an integrated model exploring the impact of knowledge, identity, and perceived risk on travel intention, to analyze its pathways and effects, to resolve the issue of mechanism, to analyze the moderating effect of past travel experience, and to answer the problem of boundary conditions. It finds that knowledge, perceived risk, and identity have a significant positive impact on travel intention; travel attitudes, subjective norms, and perceived control mediate the influence of knowledge, perceived risk, and identity on travel intention; these mechanism pathways do not always exist. The positive adjustment of past travel experiences shows that repeat visitors have a greater impact than newcomers and potential tourists.

## Introduction

**FIGURE 1:**
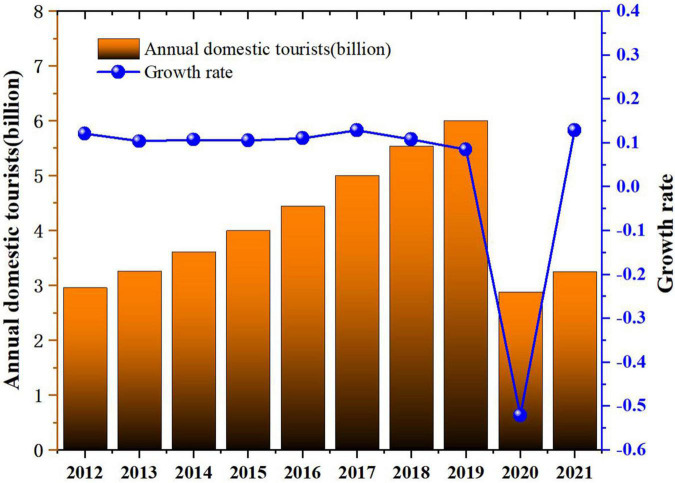
China’s annual domestic tourist volume, 2012 to 2021.

When assuming normalized circumstances, travel intention primarily arises from a combination of tourists’ personal preferences, expectations, motivations, and satisfaction, as well as destination marketing and other factors. However, existing research has tended to ignore other important influences, including subjective factors and objective environmental factors. Meanwhile, a variety and repetition of crisis events over many years have only underlined the social attributes of travel behavior. That is, whether a person engages in travel—and where to—depends not only on personal preferences, but also their knowledge regarding and ability to prevent and control tourism risks, their past travel experience, and the attitudes of other key persons or organizations regarding travel behavior at the time of decision-making. Following the initial outbreaks and rapid global spread of COVID-19, domestic and international tourism have stagnated; hospitality and other tourism-related industries have had to suspend work accordingly; no part of the industry has been unaffected (Figure 1). Despite explosive growth in the global tourism industry over the past 40 years, establishing it as one of the major engines driving global economic development, employment, and industrial transformation, the COVID-19 pandemic has hit the brakes and stalled the engines. The enormity of the pandemic’s impact on global tourism is still coming to light. This reflects not only the tight bonds within tourism production and consumption networks in the age of globalization but also the vulnerability of the industry at large. At this moment there is an urgent need for tourism scholars and industry experts to examine the relationship between tourism and the global public health crisis from a variety of perspectives. Thus far scholars have measured the impact of relevant cognitive factors on travel intention in terms of knowledge, perceived risk (Zhu and Deng, 2020), and psychological distance (Li et al., 2020). However, the literature mostly approaches behavioral intention from the perspective of individual tourists rather than groups and tends to employ one or two different theories to account for cognitive factors’ influence on travel intention. Lee and Jan (2018) believe that an integrated framework provides researchers and managers with critical insights and a more accurate grasp of the factors influencing ecotourism behavior. Thus, they construct an integrated model combining multiple theories and previously neglected factors to survey complex relations within structures, explain working mechanisms, and enhance explanatory and predictive power.

To this end, this research deepens the study of tourists’ travel intention in two aspects: integrating key factors and concepts and testing these against a moderating variable. For the former, the three factors are knowledge (as in knowledgeattitude-behavior, or KAB theory), identity (as in social identity theory, SIT), and perceived risk (as in theory of perceived risk, TPR). These are integrated to build a more comprehensive model of travel intention factors, to explore the path and effect of tourists’ action, and to answer the question of mechanism pathways. As for the latter, past travel experience is used as a moderating variable to test for changes in the relations of the abovementioned variables with varying levels of past travel experience. When considering the impact of past travel experience on current travel intention, previous papers have mainly employed binary variables to compare whether travel intention was affected by past travel experience (potential tourists and actual tourists; first-time visitors and repeat visitors) for their assessments, but the current study increases precision by dividing tourists into three graded categories—potential tourists, first-time visitors, and re-visitors—to assess the impact of past travel experience on the path of this mechanism and solve the problem of boundary conditions.

## Theoretical Background and Hypothetical Development

### Theory of Planned Behavior

The TPB is typically used to explain the relationship between attitudes and planned, intentional behavior when people have enough time to think about their attitudes. According to this theory, behavioral attitudes, subjective norms, and perceived behavioral control are the three key factors that affect behavioral intentions. Behavioral attitude is an individual’s personal evaluation of a behavior; subjective norms refer to the perception of whether important others agree with the behavior or not. These “important others” may be members of families, private networks, community organizations, work units, party organizations, and so on (Duan and Jiang, 2008). Perceived behavioral control is the sense of control an individual believes he or she has over behavior as well as the perceived difficulty of performing that behavior. Generally, tourists with better professional knowledge and who are secure in their time and monetary resources have greater perceived control and thus stronger travel intention (Zhu and Deng, 2020).

Theory of planned behavior and its extensions are often used to illustrate the mechanism behind tourists’ behavior intentions, and exhibit good explanatory and predictive power. Boguszewicz-Kreft et al. (2020) have verified the TPB model’s applicability to medical tourism and compared the differential willingness among consumers of different nationalities to use medical tourism services. Hu et al. (2021) have found that attitudes, perceived behavioral control, environmental awareness, and perceived moral obligations are significantly and positively correlated with young people’s intent for low-carbon travel behavior, while subjective norms are not. Chen et al. (2019) have applied TPB to a study of pro-environmental tourism behavior among urban residents, finding that intentions and habits are the key influential factors, while attitudes have the most significant impact on behavioral intention. Joo et al. (2020), meanwhile, have found that perceived behavioral control and subjective norms have a significant positive impact on rural tourism intention; among these two, subjective norms have a greater effect, while attitudes have no significant effect on travel intention.

### Knowledge-Attitude-Behavior

The KAB model divides human behavior into three processes: acquiring knowledge, generating belief, and forming behavior. According to this theory, attitude is the best predictor of behavior, knowledge is the basis of changes in attitude, and the degree of knowledge mastery affects the consistency of attitude and behavior. Thus tourism knowledge is the key to the development of attitudes and travel behavior, but this “knowledge” is different from knowledge in the objective sense; rather, it is an abstracted perception of knowledge that directly affects tourists’ psychology and decision-making practices, as through the arousal of confidence and willingness to act (Quintal et al., 2010; Sharifpour et al., 2014). Zhu and Deng (2020) define such knowledge perception as a tourist’s mental assessment of his or her ability to identify and understand the risks of tourism and COVID-19, the danger these pose to humans and the tourism industry, as well as countermeasures and other related issues. Psychologically, having more knowledge can increase an individual’s personal control over uncertain scenarios (Zhang et al., 2021). When travelers think that they have more knowledge than others, and thus a greater ability to prevent and control risks, they are more likely to participate in tourism activities (Tassiello and Tillotson, 2020). Therefore, considering the above analysis, this study puts forth the following hypotheses:

**H1:** Knowledge (a: knowledge of tourism; b: knowledge of COVID-19) has a direct and indirect positive impact on travel intention through attitudes.

**H2:** Knowledge has a direct and indirect positive impact on travel intention through subjective norms.

**H3:** Knowledge has a direct and indirect positive impact on travel intention through perceived behavioral control.

### Social Identity Theory

Social identity theory focuses on individual behavior in group settings. It suggests that group belonging (regardless of that group’s size or distribution) is, to a large extent, an individual mental state, but one that is totally distinct from that person’s independent mental state outside of the group setting. Belonging to a group gives one social identity, or a shared collective answer to the question “who am I?”, along with a set of behaviors appropriate to that identity (Abrams and Hogg, 2006). According to SIT, “identity” includes both self-identity, social identity, etc.; while self-identity is an individual’s perception of self-consistency and continuity (Erikson, 1968); social identity is an individual’s awareness of belonging to a specific social group (Tajfel, 1982). Such identification can work to depersonalize an individual’s self-perception and social actions.

In their research on the relationship between identity and past travel experience, Gieling and Ong (2016) find that attitudes and behaviors are affected by important social relationships. Likewise, Forsyth et al. (2015) suggest that a sense of community can influence individual behavior. Canovi (2019), meanwhile, finds that winemakers’ identities are moderated by the local community, which ultimately affects their attitudes toward diversified tourism development. It is clear that travel intention is inseparable from larger social contexts. Researchers must move beyond understandings of individual intention and formulate their studies to account for communities’ collective consciousnesses, individual respect for collective interests, and the association of individual behavior with important social members or organizations. In light of this, the following hypotheses are put forward:

**H4:** Tourism self-identity has a direct and indirect positive impact on travel intention through attitudes.

**H5:** Tourism self-identity has a direct and indirect positive impact on travel intention through subjective norms.

**H6:** Tourism self-identity has a direct and indirect positive impact on travel intention through perceived behavioral control.

### Theory of Perceived Risk

The basic principles of TPR are based on the theory of bounded rationality and satisfaction. Bauer (1960) believes that when consumers make decisions, they do not seek to “maximize utility” as economists call it, but to minimize the associated risk. According to TPR, perceived risk is one’s expectation that he or she may suffer losses. This subjective take is important because if a tourist does not perceive risk, it may not affect his or her travel decisions; conversely, even in the absence of objective risk, a tourist’s perception of its presence may affect decision-making nevertheless (Khan et al., 2019).

Initial research on perceived risk tended to suggest that the greater tourists’ perceived risk—in terms of time, economy, physical and mental health, etc.—the more likely they are to lower their travel intention in avoidance of said risk (Fischer et al., 1991; Roehl and Fesenmaier, 1992; Zhu and Deng, 2020). As research has deepened, perceived risk’s positive effect on behavioral intention has received much attention. Scholars have found that perceived risk can enhance public attention to risk, crisis awareness, risk recognition and understanding, the ability to interpret risk information in a calm manner, to participate in discussions on risk, and to form reasonable perceptions and attitudes in relation to risk—important outcomes beneficial to the reduction of perceived risk and formation of positive behavioral attitudes (Cui et al., 2016). And, as novel phenomena inevitably inspire some people’s curiosity, a certain level of perceived risk may actually inspire more adventurous attitudes and a willingness to face challenges. Reisinger and Mavondo (2005) find that backpackers have higher risk tolerance than group tourists and will seek out moderately high levels of risk to increase the excitement of travel. Vespestad et al. (2019), meanwhile, propose that higher risk perception in adventure tourism is a point of attraction for its potential consumers. Thus, considering that perceived risk can also stimulate a person’s intent to travel, this study puts forth the following hypotheses:

**H7:** Perceived risk has a direct and indirect positive impact on travel intention through attitudes.

**H8:** Perceived risk has a direct and indirect positive impact on travel intention through subjective norms.

**H9:** Perceived risk has a direct and indirect positive impact on travel intention through perceived behavioral control.

### Moderating Role of Past Travel Experience

Past travel experience refers to individuals’ prior instances of personal participation in tourism activities. Such experience can increase willingness to revisit (Roehl and Fesenmaier, 1992; Sönmez and Graefe, 1998; Vespestad et al., 2019). When it comes to behavioral intention, past travel experience has greater explanatory power than other variables in TPB, and when it comes to tourism behavior, in particular, past travel experience is considered a key determinant (Ajzen, 2002). Tourists with different past travel experiences will differ significantly in cognitive levels and emotional attitudes, so past travel experience is often regarded as an important moderating factor (Hammond et al., 1998; Ajzen, 2002). Deutsch and Krauss (1965) find that compared to indirect experience, personal experience can affect more consistency in attitude and behavior. For a more specific illustration, Beldona et al. (2005) find that earlier (i.e., more experienced) users of online travel sites are more likely to purchase online travel products than later users. Murodjon et al. (2021) also found that past personal experience had a significant impact on tourists’ behavioral intention in their study of Uzbekistan’s Silk Road tourism. Kim and Chen (2021), when comparing two groups of interviewees, find that those with firsthand experience expressed higher destination loyalty and stronger behavioral intention. In view of these findings, this study puts forward the following hypotheses:

**H10:** Past travel experience moderates the impact of (a) knowledge, (b) tourism self-identity, (c) perceived risk, (d) attitude, (e) subjective norms, and (f) perceived behavioral control on travel intention.

**H11:** Past travel experience moderates the impact of (a) knowledge, (b) tourism self-identity, and (c) perceived risk on attitude.

**H12:** Past travel experience moderates the impact of (a) knowledge, (b) tourism self-identity, and (c) perceived risk on subjective norms.

**H13:** Past travel experience moderates the impact of (a) knowledge, (b) tourism self-identity, and (c) perceived risk on perceived behavioral control.

Lepp and Gibson (2003) indicate past travel experience as an important factor affecting tourists’ perceived risk. Likewise, Fuchs and Reichel (2011) find differences in risk perception between first-time and repeat visitors. As repeat visitors generally have more experience preventing and controlling tourism risks, their risk perception is lowered, and it is easier for them to form positive travel attitudes. At the same time, repeat visitors’ perceived control has a more pronounced effect on their travel intention; experienced tourists will even ignore the risks involved. They are also more familiar with the variety of travel activities and applicable precautions, more inclined to support the unified management of communities and destinations, and feel more in control of their behavior during travel. Accordingly, it may be deduced that the mediating role of attitudes, subjective norms, and perceived behavioral control on knowledge, tourism self-identity, and perceived risk’s impact on travel intention may be yet further regulated by past travel experience. Therefore, it is proposed that:

**H14:** Past travel experience moderates the mediation of (a) attitudes, (b) subjective norms, and (c) perceived behavioral control between knowledge and travel intention.

**H15:** Past travel experience moderates the mediation of (a) attitudes, (b) subjective norms, and (c) perceived behavioral control between tourism self-identity and travel intention.

**H16:** Past travel experience moderates the mediation of (a) attitudes, (b) subjective norms, and (c) perceived behavioral control between perceived risk and travel intention.

**FIGURE 2:**
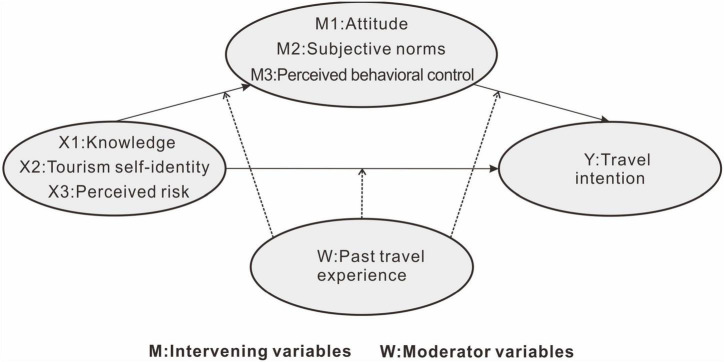
Conceptual diagram of a final integrated model.

By combining these hypotheses, this study proposes a comprehensive theoretical model (Figure 2).

## Materials and Methods

### Questionnaire Design and Variable Measurement

The main body of the questionnaire utilizes the 5-point Likert scale (1 = strongly disagree, 5 = strongly agree). To ensure the reliability and validity of results, its design draws upon well-established scales, with adjustments to fit the specific context and goals of the study. With reference to Zhu and Deng (2020), knowledge is measured in two aspects—knowledge of tourism, and knowledge of COVID-19—across eight items, while the perceived risk is measured across 10 items and in four dimensions: physical, cost, performance, and equipment risk. Tourism self-identity, subjective norms, and perceived behavioral control draw on the well-established scales of Lee and Jan (2018) and are measured by four to five items each, while travel intention is measured by three items. As categorical variables cannot accurately measure the degree of impact the environment exerts during a certain experience, this study selects graded variables to measure past travel experience, dividing the respondents into three categories—potential tourists, first-time visitors, and repeat visitors—to analyze the moderating effect of past travel experience. A pre-test of reliability finds that after deleting the item “(KP1) I know the original cause of COVID-19”, the reliability coefficient improves significantly; this item was thus deleted during the revision of the questionnaire (Table 1).

**TABLE 1:** Results of exploratory factor analysis (EFA).

Items	Factor loading								Communality
	PR	SN	KC	KT	ATT	TID	PBC	TI	
KC2: I know about the harm caused by COVID-19	−0.04	0.02	**0.90**	0.10	0.16	0.03	0.02	0.00	0.85
KC3: I know about the length of COVID-19’s incubation period	0.00	0.03	**0.91**	0.09	0.14	0.05	0.03	0.05	0.86
KC4: I know about the current affected range for COVID-19	−0.04	−0.02	**0.89**	0.13	0.07	0.10	0.07	0.00	0.84
KC5: I know about preventive measures for COVID-19	−0.03	0.07	**0.90**	0.10	0.14	0.02	0.04	0.01	0.85
ATT1: Participating in tourism can enhance my quality of life	0.11	0.14	0.14	0.09	**0.87**	0.13	0.09	0.15	0.87
ATT2: Participating in tourism can help improve my job performance	0.02	0.14	0.12	0.09	**0.84**	0.18	0.13	0.02	0.79
ATT3: Participating in tourism can help me gain knowledge	0.12	0.22	0.17	0.13	**0.87**	0.12	0.10	0.10	0.89
ATT4: Participating in tourism contributes to my physical and mental health	0.16	0.17	0.19	0.12	**0.84**	0.13	0.11	0.19	0.88
PR1: I may get sick on the trip, for example, COVID-19	**0.75**	−0.01	0.00	0.11	0.05	0.03	0.06	−0.01	0.59
PR2: Tourist attractions have poor infrastructure	**0.84**	0.04	−0.02	0.03	0.02	−0.05	0.01	−0.01	0.71
PR3: Tourist attractions have poor sanitation	**0.87**	0.02	−0.04	0.03	−0.02	0.03	0.02	−0.04	0.76
PR4: Traffic is inconvenient at tourist spots	**0.88**	−0.03	−0.02	0.06	−0.04	0.04	0.05	0.02	0.78
PR5: Actual travel costs will exceed expectations during a trip	**0.83**	0.08	−0.04	−0.01	0.07	0.07	0.02	0.01	0.71
PR6: Tourism will waste much time on the road	**0.87**	−0.01	−0.01	0.01	0.10	0.03	0.02	0.12	0.78
PR7: Due to quarantine measures, travel will take more time	**0.77**	−0.08	0.04	0.13	0.13	0.05	0.07	0.15	0.67
PR8: Due to travel restrictions, certain services cannot be provided as planned	**0.81**	−0.05	−0.02	0.15	0.13	0.06	0.09	0.17	0.73
PBC1: Enough time to participate in tourism	0.08	0.20	−0.04	0.11	0.11	0.06	**0.78**	0.15	0.71
PBC2: Enough money to participate in tourism	0.03	0.13	0.04	0.11	0.09	0.14	**0.81**	0.12	0.73
PBC3: Enough information to participate in tourism	0.08	0.20	0.02	0.15	0.11	0.19	**0.83**	0.13	0.82
PBC4: Enough knowledge to participate in tourism	0.12	0.17	0.16	0.22	0.09	0.10	**0.74**	0.00	0.69
KT1: I am concerned about travel information	0.18	0.12	0.05	**0.82**	0.16	0.16	0.08	0.06	0.78
KT2: I know about the causes of tourism risks	0.11	0.12	0.13	**0.88**	0.08	0.19	0.17	0.08	0.89
KT3: I know about the consequences of tourism risks	0.16	0.11	0.12	**0.88**	0.10	0.14	0.15	0.07	0.88
KT4: I know about the solutions to tourism risks	0.02	0.13	0.20	**0.83**	0.09	0.14	0.22	0.04	0.83
TID1: I share an interest in tourism with other members of my community	0.14	0.13	0.04	0.21	0.16	**0.78**	0.10	0.11	0.74
TID2: I engage in tourism along with other members of my community	0.00	0.21	−0.02	0.08	0.08	**0.82**	0.20	0.01	0.78
TID3: The members of my community are very important to me	−0.02	0.19	0.11	0.13	0.10	**0.85**	0.15	0.05	0.82
TID4: I consider myself a member of my community	0.10	0.16	0.10	0.22	0.21	**0.76**	0.03	0.11	0.73
TI1: When the destination is safe, I am willing to participate in tourism	0.07	0.20	0.03	0.21	0.18	0.08	0.13	**0.69**	0.63
TI2: I am willing to participate in tourism within a year after the destination is	0.11	0.38	0.00	0.09	0.13	0.06	0.14	**0.80**	0.84
declared safe									
TI3: I am likely to participate in tourism within a year after the destination is	0.18	0.30	0.03	−0.04	0.12	0.13	0.16	**0.80**	0.82
declared safe									
SN1: My teachers approve of my participation in tourism	−0.05	**0.83**	0.02	0.12	0.15	0.20	0.18	0.10	0.81
SN2: My parents approve of my participation in tourism	−0.07	**0.83**	0.02	0.05	0.10	0.14	0.18	0.18	0.79
SN3: My colleagues approve of my participation in tourism	0.00	**0.86**	−0.02	0.11	0.12	0.20	0.13	0.14	0.84
SN4: My friends approve of my participation in tourism	0.05	**0.85**	0.05	0.07	0.15	0.14	0.10	0.19	0.81
SN5: The government approves of my participation in tourism	−0.01	**0.80**	0.06	0.16	0.16	0.06	0.19	0.20	0.77
Eigen value (Unrotated)	10.16	5.41	3.60	2.52	1.98	1.73	1.65	1.17	–
% of Variance (Unrotated)	28.23	15.04	10.00	7.01	5.49	4.81	4.58	3.26	–
Cumulative% of Variance (Unrotated)	28.23	43.27	53.26	60.27	65.76	70.58	75.16	78.41	–
Eigen value (Rotated)	5.728	4.172	3.483	3.38	3.375	3.015	2.952	2.124	–
% of Variance (Rotated)	15.91	11.59	9.68	9.39	9.38	8.37	8.20	5.90	–
Cumulative% of Variance (Rotated)	15.91	27.50	37.18	46.56	55.94	64.31	72.51	78.41	–
KMO	0.88								–
Bartlett’s Test of Sphericity	11,523.84								–
df	630								–
*p* value	0.000								–

*PR, perceived risk; SN, subjective norms; KC, knowledge of COVID-19; KT, knowledge of tourism; ATT, attitudes; TID, tourism self-identity; PBC, perceived behavioral control; TI, travel intention. Bold values indicates the absolute value of factor loading is greater than the threshold.*

### Survey Sampling and Data Collection

The sample size formula designed by Yamane (1967) is as below.


$$n=\frac{N}{1+N\,{\left(e\right)}^{2}}$$

Where *n* represents sample size, *N* stands for the population, and *e* represents the precision level. Usually, e was at a 95% confidence level. In this study, in accordance with the figure 3.25 billion domestic person-trips for China in 2021, the formula yielded an *n* of 399.9 as the minimum acceptable sample size.

This study, using convenience sampling, collected data using the professional survey application ‘‘Questionnaire Star’’,^1^ whose paying customers to cover more than 30,000 companies and 90% of universities in China. The official questionnaire was open from 26 December 2020 to 26 January 2021. After applying the “Questionnaire Recommendation Service” through Questionnaire Star, the online system randomly invited people from its 2.6-million-sample database to fill in their responses. Beyond that, members of the research team and the surrounding community were invited to respond to the survey as well—via weblink, QR code, or WeChat message.

The average time for each questionnaire is 487 s. The questionnaires with an answering time of less than 1 min or a missing proportion of more than 70% are considered invalid questionnaires. After the invalid questionnaires were deleted, a total of 405 valid questionnaires were obtained. Among the valid questionnaires, 69.8% of respondents are men and 30.2% are women; in terms of age structure, the largest group is 18–25 years (67.57%), followed by 26–30 years (18.81%) and 31–40 years (10.64%); as for educational attainment, the most common level is college or post-secondary professional schooling (55.56%), followed by high school or vocational schooling (25.68%), then masters or doctoral study (15.8%). Respondents come from 89 cities in 27 provinces and cover 15 occupations in 24 industries. Potential tourists, first-time visitors, and repeat visitors account for 20.15, 13.18, and 66.67% of respondents, respectively. In the first year of COVID (23 January 2020 to 26 January 2021), 80.3% of tourists did not participate in tourism, while 19.7% of tourists did participate in tourism activities.

### Analysis Methods

For data analyses, the SPSSAU data scientific analysis platform (https://spssau.com/) developed by Changsha Ranxing Information Technology Co., Ltd., location in Changsha, China, was used to run reliability and validity tests, descriptive statistics, and correlation coefficient checks. Building on that, hierarchical regression analysis was employed to test the main effect, mediated effect, and moderating effect, while a bias-corrected non-parametric percentile method (bootstrapping) was used to test for mediating and moderated mediating effects.

## Results

### Reliability and Validity Analysis

First is index classification analysis. Thirty-six items were enriched through exploratory factor analysis (EFA) and rotated with maximum variance rotation. During factor analysis, KMO values were 0.88 > 0.6, indicating that the data can be used for factor analysis (Kaiser, 1974). The final condensation into eight factors is shown in Table 1. Cumulative variance is 78.41%, meaning that these eight factors are able to extract 78.41% of information from the total 36 items; variance (rotated)—that is, the amount of information extracted—for the eight factors following rotation are 15.91, 11.59, 9.68, 9.39, 9.38, 8.37, 8.2, and 5.9, respectively. This distribution is sufficiently uniform to demonstrate the comprehensive soundness of factor analysis results. Moreover, the 36 items fit well with professional expectations. Integrating the congruence between factors and analysis items, the eight condensed factors are ultimately named: “perceived risk,” “subjective norms,” “knowledge of COVID-19,” “knowledge of tourism,” “attitudes,” “tourism self-identity,” “perceived behavioral control,” and “travel intention.”

The second is the reliability test, which employs reliability analysis. The data involves eight dimensions: perceived risk, subjective norms, knowledge of COVID-19, knowledge of tourism, attitudes, tourism self-identity, perceived behavioral control, and travel intention. Cronbach’s alpha (α) measures the quality of data reliability. If the value of α is higher than 0.8, the data is highly reliable; if α is between 0.7 and 0.8, reliability is good; a value between 0.6 and 0.7 indicates acceptable reliability, and a value of 0.6 or below indicates poor reliability (Eisinga et al., 2013). As shown in Table 2, the questionnaire’s total score is 0.921 and all eight dimensions have a Cronbach’s alpha value higher than 0.8, with the lowest dimension scoring 0.831. Data reliability is thus of high quality, and the data is considered credible and true.

**TABLE 2:** Results of reliability analysis.

Factor	Item	Cronbach’s alpha
Knowledge of tourism	4	0.938
Knowledge of COVID-19	4	0.935
Tourism self-identity	4	0.891
Perceived risk	8	0.939
Attitudes	4	0.942
Subjective norms	5	0.935
Perceived behavioral control	4	0.884
Travel intention	3	0.831
Total reliability	36	0.921

The third is the convergent validity analysis. Confirmatory factor analysis (CFA) is conducted for a total of eight factors and 36 analysis items, as shown in Table 2. The effective sample size is 405—more than ten times the number of analysis items, and within the moderate range (Everitt, 1975). The study finds that all measurement items are significant to the.001 level (*p* < 0.001). The scale of the study is represented by eight condensed factors. As shown in Table 3, the factors’ average variance extracted (AVE) values are all greater than 0.6, with a minimum value of 0.634, significantly exceeding the standard of 0.5, while composite reliability (CR) values are all greater than.8, significantly exceeding the respective standard of 0.7. The study scale thus demonstrates excellent convergent validity (Gim Chung Ruth et al., 2004). Moreover, the standardized factor loading coefficients for all 36 items corresponding to the eight factors, given in Table 4, are greater than or equal to 0.7, comprehensively indicating the excellent convergent validity of scale data in this study.

**TABLE 3:** Results of confirmatory factor analysis.

Factor	AVE	CR
Knowledge of tourism	0.786	0.936
Knowledge of COVID-19	0.794	0.939
Tourism self-identity	0.666	0.888
Attitudes	0.800	0.941
Subjective norms	0.742	0.935
Perceived behavioral control	0.634	0.873
Perceived risk	0.655	0.938
Travel intention	0.677	0.858

**TABLE 4:** Standardized factor loading.

Factor	Item	Std. estimate	Factor	Item	Std. estimate	Factor	Item	Std. estimate
Knowledge of tourism	KT1	0.818	Perceived behavioral control	PBC1	0.78	Attitudes	ATT1	0.872
	KT2	0.876		PBC2	0.812		ATT2	0.839
	KT3	0.881		PBC3	0.828		ATT3	0.865
	KT4	0.833		PBC4	0.744		ATT4	0.843
Knowledge of COVID-19	KC2	0.901	Perceived risk	PR1	0.754	Subjective norms	SN1	0.828
	KC3	0.906		PR2	0.84		SN2	0.828
	KC4	0.894		PR3	0.867		SN3	0.857
	KC5	0.901		PR4	0.876		SN4	0.845
Tourism self-identity	TID1	0.779		PR5	0.833		SN5	0.795
	TID2	0.824		PR6	0.869	Travel intention	TI1	0.7
	TID3	0.849		PR7	0.772		TI2	0.798
	TID4	0.758		PR8	0.807		TI3	0.796

The fourth is discriminant validity testing. Discriminant validity is measured by comparing the square root of AVE—representing that factor’s convergence—with factors’ correlation coefficients, which express the degree of correlation. If a factor’s convergence is very strong (significantly stronger than the “correlation coefficient between this factor and other factors”), then it is considered to have discriminant validity. In this study, Pearson correlation analysis is performed first to determine the interfactor correlation coefficients. Next, the square root of each factor’s AVE value is compared against the interfactor correlation coefficients. The results of this analysis are given in Table 5. The square roots of AVE for “knowledge of COVID-19” is 0.891, which is greater than the correlation coefficients between “knowledge of COVID-19” and all seven other factors (the highest is 0.547); similarly, the square roots of AVE for “knowledge of tourism” is 0.887, which is greater than all its correlation coefficients with other factors (the highest is 0.627); indeed, all eight factors have AVE root values that are higher than their interfactor correlation coefficients. Therefore, the study’s scale data has good discriminant validity (Gim Chung Ruth et al., 2004).

**TABLE 5:** Means, standard deviations, and correlation coefficients for study variables.

	Means	Standard deviation	KC	KT	TID	PR	ATT	SN	PBC	TI
KC	3.939	0.780	**0.891**							
KT	3.435	0.710	0.300***	**0.887**						
TID	3.365	0.628	0.253***	0.627***	**0.816**					
PR	3.384	0.714	0.079	0.295***	0.250***	**0.809**				
ATT	3.819	0.676	0.547***	0.390***	0.457***	0.317***	**0.894**			
SN	3.290	0.672	0.130**	0.382***	0.500***	0.133**	0.404***	**0.861**		
PBC	3.282	0.612	0.180***	0.491***	0.487***	0.479***	0.421***	0.460***	**0.796**	
TI	3.283	0.682	0.151**	0.428***	0.559***	0.307***	0.446***	0.712***	0.505***	**0.823**

*KT, knowledge of tourism; KC, knowledge of COVID-19; TID, tourism self-identity; PR, perceived risk; ATT, attitudes; SN, subjective norms; PBC, perceived behavioral control; TI, travel intention. Bold numbers are the square roots of average variance extracted values.*

**p < 0.01.

***p < 0.001.

### Multicollinearity, Autocorrelation, and Normality Tests

Linear regression analysis is used to assess how perceived risk, subjective norms, knowledge of COVID-19, knowledge of tourism, attitudes, tourism self-identity, and perceived behavioral control relate to “travel intention”. The study finds that the model passes the *F*-test (*p* < 0.001); in other words, the model is meaningful, and at least one of the seven factors will have an impact on travel intention. As the model’s R^2^ value is 0.79, these seven factors should explain 79% of the variation in travel intention. In addition, the model’s multicollinearity test results in a maximum VIF value of 1.79, which means that all factors’ VIF values are less than 5, indicating that there is no collinearity, and the model is satisfactory (Hauke and Kossowski, 2011).

The Kolmogorov-Smirnov test is used to analyze the data’s distribution normality. It finds the skewness coefficient’s absolute value to fall between 0.12 and 1.05 and the kurtosis coefficient’s absolute value to fall between 0.12 and 2.15—well below the critical values of 3 and 8, respectively. The sample thus passes the normality test (Drezner et al., 2010).

### Descriptive Statistical Analysis

Pearson correlation analysis is used to verify correlation and the strength of the relationships between variables. The study finds a significant positive correlation between the following factors (Table 5): knowledge of tourism and attitudes (*r* = 0.39, *p* < 0.001), knowledge of COVID-19 and attitudes (*r* = 0.55, *p* < 0.001), knowledge of tourism and subjective norms (*r* = 0.38, *p* < 0.001), knowledge of COVID-19 and subjective norms (*r* = 0.13, *p* < 0.01), knowledge of tourism and perceived behavioral control (*r* = 0.49, *p* < 0.001), knowledge of COVID-19 and perceived behavioral control (*r* = 0.18, *p* < 0.001), tourism self-identity and attitudes (*r* = 0.46, *p* < 0.001), tourism self-identity and subjective norms (*r* = 0.5, *p* < 0.001), tourism self-identity and perceived behavioral control (*r* = 0.49, *p* < 0.001), perceived risk and attitudes (*r* = 0.32, *p* < 0.001), perceived risk and perceived behavioral control (*r* = 0.48, *p* < 0.001), knowledge of tourism and travel intention (*r* = 0.43, *p* < 0.001), knowledge of COVID-19 and travel intention (*r* = 0.15, *p* < 0.01), tourism self-identity and travel intention (*r* = 0.56, *p* < 0.001), perceived risk and travel intention (*r* = 0.31, *p* < 0.001), attitudes and travel intention (*r* = 0.45, *p* < 0.001), subjective norms and travel intention (*r* = 0.71, *p* < 0.001), perceived behavioral control and travel intention (*r* = 0.51, *p* < 0.001). These results provide preliminary evidence for subsequent hypothesis testing.

### Model Comparison Analysis

The study selects the most commonly used fitting indexes such as GFI, NNFI, CFI, RMR, RMSEA, etc. in order to analyze the fit of the model. As shown in Table 6, compared with the KAB, SIT and TPB models, the integrated model has better fitting conditions and more reliable results.

**TABLE 6:** The model indices of the four theoretical models.

Theoretical models	^χ2^	*df*	^χ2^/*df*	GFI	NNFI	CFI	RMR	RMESA		Δχ^2^	Δ*df*	p
	
	–	–	<3	>0.9	>0.9	>0.9	<0.10	<0.10				
IM	415.43	150	2.77	0.93	0.97	0.98	0.05	0.07				
KAB	192.96	20	9.65	0.89	0.91	0.93	0.08	0.16	IM-KAB	222.47	130	0.000
SIT	49.68	5	9.94	0.95	0.94	0.97	0.05	0.15	IM-SIT	365.75	145	0.000
TPB	39.74	2	19.87	0.95	0.90	0.97	0.06	0.21	IM-TPB	375.69	148	0.000

*IM, integrated model.*

According to Jöreskog and Sörbom (1996), χ^2^ can test whether there is a statistically significant difference between the two competing models in their ability to explain covariance. Therefore, the study compares three competitive models (TPB model, SIT model, and KAB model) to determine the optimal model (integrated model). The fitting indexes of the four theoretical models in Table 6 show that the integrated model is better. The explanatory power of the integrated model and the competitive model showed a significant difference (*p* < 0.001).

### Hypothesis Testing

#### Main Effect Test

After controlling for demographic and travel behavior characteristics—such as gender, age, marital status, educational attainment, industry, occupation, tourist origin, etc., regression analysis is used to verify the influence of independent variables on dependent variables. The study finds significant positive influences on travel intention for the following factors: knowledge (second-order) (β = 0.32, *p* < 0.001), knowledge of tourism (β = 0.35, *p* < 0.001), knowledge of COVID-19 (β = 0.11, *p* < 0.05), perceived risk (β = 0.24, *p* < 0.001), tourism self-identity (β = 0.58, *p* < 0.001). This means that knowledge, perceived risk, and tourism self-identity can increase travel intention.

#### Mediation Effect Test

After controlling for demographic and behavioral characteristics, the Bootstrap sampling method is used to test the mediating effect. The Bootstrap test for the sampling method refers to whether the 95% CI for the regression coefficient a*b contains the number 0; if it does not include the number 0, it means a mediating effect is present; if it does include the number 0, then there is no mediating effect. The results after sampling 5,000 times are shown in Table 7. In total there are five independent variables, three mediator variables, and 15 mediation paths—among these, nine paths are fully mediated; four paths are partially mediated, and two paths are not significantly mediated.

**TABLE 7:** Results of mediation analysis.

*Totaleffect*		*a*	*b*	Mediation effect	95% BootCI	Direct effect	Conclusion	Effect size (%)
TI←ATT←K	0.32***	0.54***	0.11**	0.06	0.01∼0.10	0.01	Full mediation	100
TI←SN←K	0.32***	0.30***	0.60***	0.18	0.0∼0.23	0.01	Full mediation	100
TI←PBC←K	0.32***	0.36***	0.20***	0.07	0.02∼0.11	0.01	Full mediation	100
TI←ATT←KT	0.35***	0.38***	0.11**	0.04	0.01∼0.08	0.02	Full mediation	100
TI←SN←KT	0.35***	0.34***	0.60***	0.21	0.12∼0.29	0.02	Full mediation	100
TI←PBC←KT	0.35***	0.40***	0.19***	0.08	0.02∼0.13	0.02	Full mediation	100
TI←ATT←KC	0.11*	0.34***	0.12**	0.04	0.01∼0.08	–0.02	Full mediation	100
TI←SN←KC	0.11*	0.10*	0.60***	0.06	0.01∼0.13	–0.02	Full mediation	100
TI←PBC←KC	0.11*	0.12**	0.20***	0.02	0.00∼0.06	–0.02	Full mediation	100
TI←ATT←TID	0.58***	0.45***	0.07	0.03	−0.00∼0.07	0.26***	Not significant	0
TI←SN←TID	0.58***	0.45***	0.53***	0.24	0.16∼0.31	0.26***	Partial mediation	41.17
TI←PBC←TID	0.58***	0.36***	0.14**	0.05	0.01∼0.10	0.26***	Partial mediation	8.78
TI←ATT←PR	0.24***	0.20***	0.10**	0.02	0.00∼0.05	0.13***	Partial mediation	8.96
TI←SN←PR	0.24***	0.07	0.63***	0.05	−0.04∼0.13	0.13***	Not significant	0
TI←PBC←PR	0.24***	0.35***	0.12*	0.04	0.01∼0.11	0.13***	Partial mediation	17.94

*K, knowledge; KT, knowledge of tourism; KC, knowledge of COVID-19; TID, tourism self-identity; PR, perceived risk; ATT, attitudes; SN, subjective norms; PBC, perceived behavioral control; TI, travel intention.*

*p < 0.05.

**p < 0.01.

***p < 0.001.

The 95% CIs corresponding to the mediating effect (on travel intention) of attitudes, subjective norms, and perceived behavioral control in knowledge (second-order), knowledge of tourism, and knowledge of COVID-19 do not contain 0, indicating a significant mediation path, and the effect sizes were all 100%. This means that the effect of knowledge on travel intention works through attitudes, subjective norms, and perceived behavioral control. That is to say, the findings support Hypotheses 1-3: knowledge can both directly influence travel intention and indirectly promote travel intention when mediated by attitudes, subjective norms, and perceived behavioral control.

The 95% CIs respectively corresponding to the mediating effects of subjective norms and perceived behavioral control in tourism self-identity’s effect on travel intention are [0.16,0.31] and [0.01,0.1], not containing 0, indicating a significant mediating effect. This validates Hypotheses 5 and 6: tourism self-identity can both directly influence travel intention and indirectly promote travel intention through the mediation of subjective norms and perceived behavioral control. In the two groups, the indirect effects of subjective norms and perceived behavioral control are 41.17 and 8.78%, respectively.

The 95% CIs respectively corresponding to the mediating effect of attitudes and perceived behavioral control in perceived risk’s effect on travel intention are [0.00,0.05] and [0.01,0.11], not containing 0, indicating a significant mediating effect. Thus Hypotheses 7 and 9 hold: that perceived risk can both directly influence travel intention and indirectly promote travel intention through the mediation of attitudes and perceived behavioral control. In the two groups, the indirect effects of attitudes and perceived behavioral control are 8.96 and 17.94%, respectively.

As for the two remaining paths, however, (TI←ATT←TID and TI←SN←PR), the 95% CIs are [−0.00,0.07] and [−0.04,0.13], and contain the number 0, meaning that the mediating effect is not significant. Thus perceived risk does not affect travel intention indirectly via subjective norms, and tourism self-identity does not affect travel intention indirectly via attitudes; Hypotheses 4 and 8 do not hold.

#### Test of the Moderating Effect

After centering the independent and moderator variables and controlling for demographic and travel behavior characteristics, the moderating effect is tested. This analyzes the effect of independent variables on dependent variables and whether or not the moderator comes into play—that is, when the level of past travel experience varies, whether there is a significant difference in the magnitude of influence.

**FIGURE 3:**
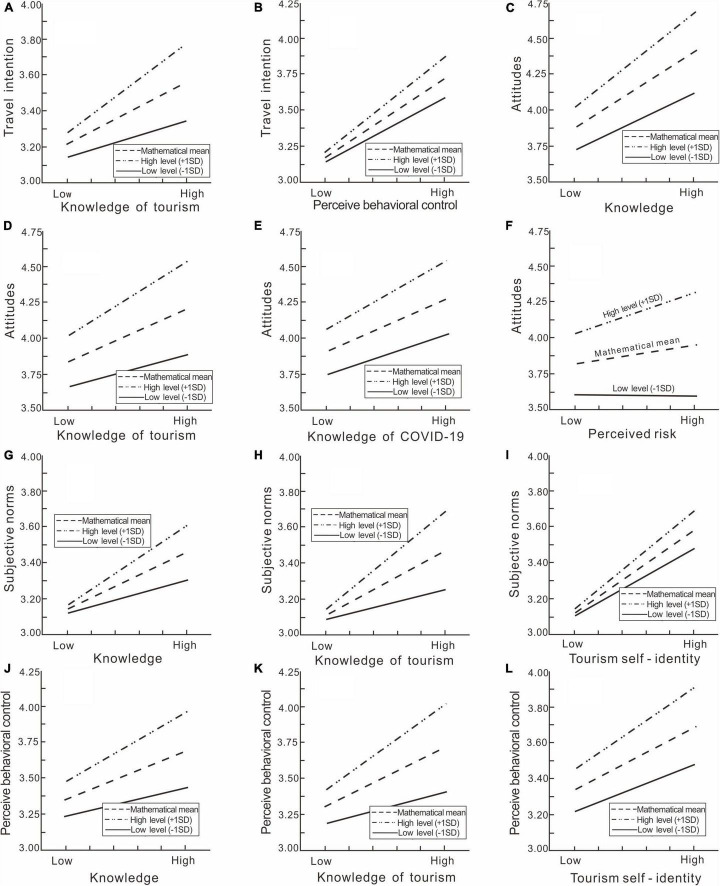
**(A,B)** The moderating effect of past travel experience on travel intention. **(C–F)** The moderating effect of past travel experience on attitudes. **(G–I)** The moderating effect of past travel experience on subjective norms. **(J–L)** The moderating effect of past travel experience on perceived behavioral control.

The study finds that when the level of past travel experience varies, the impact of knowledge, knowledge of COVID-19, tourism self-identity, perceived risk, attitude, and subjective norms on travel intention are the same (*p* > 0.05). Therefore, H10b–H10e do not hold, the effects of knowledge, knowledge of COVID-19, tourism self-identity, perceived risk, attitude, and subjective norms on travel intention were completely consistent regardless of the subject’s status as a first-time visitor, repeat visitor, or potential tourist. Generally speaking, the influence that knowledge exerts over travel intention is unperturbed by the factor of past travel experience; however, this is not always the case. While past travel experience does not interfere with the effect of knowledge of COVID-19 on travel intention, it does interfere with the effect of knowledge of tourism on travel intention (*p* = 0.001). The strength of interference varies as follows: repeat visitors > first-time visitors > potential tourists (Figure 3A). Therefore, H10a holds in part. As shown in Figure 3B, the effect of perceived behavioral control on travel intention is positively moderated by the interference of past travel experience (*p* = 0.036). The more travel experience, the greater the effect. That is, repeat visitors > first-time visitors > and potential tourists. H10f is therefore supported.

As shown in Figures 3C–F, the impact of knowledge (second-order) (*p* = 0.013), knowledge of tourism (*p* = 0.000), knowledge of COVID-19 (*p* = 0.022), and perceived risk (*p* = 0.002) on attitude are significantly different, indicating past travel experience regulates the impact of these factors on attitude. The richer the past travel experience, the more positive the attitude. H11a and H11c thus hold. When the level of past travel experience varies, the impact of tourism self-identity on attitude is the same (*p* > 0.05). H11b is not supported.

As shown in Figures 3G–I, the impact of knowledge (second-order), knowledge of tourism, and tourism self-identity on subjective norms vary with the level of past travel experience. When past travel experience is richer, the respective impact of the three abovementioned factors on subjective norms is greater. H12b thus holds while H12c is not supported. H12a partially holds. Past travel experience tends to interfere with the effect of knowledge on subjective norms, but not invariably; it moderates the effect of knowledge of tourism (as shown in Figure 3H: repeat visitors > first-time visitors > potential tourists) but does not moderate the effect of knowledge of COVID-19 on subjective norms (*p* > 0.05).

As shown in Figures 3J–L, the impact of knowledge (second-order), knowledge of tourism, and tourism self-identity on perceived behavioral control vary with the level of past travel experience. When past travel experience is richer, the impact of the three abovementioned factors on perceived behavioral control is greater. H13b thus holds while H13c is not supported. H13a holds in part. Past travel experience generally does moderate the effect of knowledge on perceived behavioral control, but not always; while the effect of knowledge of tourism is affected by past travel experience (as shown in Figure 3K: repeat visitors > first-time visitors > potential tourists), the effect of knowledge of COVID-19 on behavioral control is not thus affected (*p* > 0.05).

#### Moderated Mediation Model Test

In the moderated mediation model, the independent variable affects the dependent variable through a moderator variable such that the process of mediation is moderated by it. the moderated mediation model is used to analyze whether there are significant differences in the mediating effect with varying levels of a moderator variable. The test results for this study are given in Table 8. When there is a high level of past travel experience, the mediating effect of knowledge (second-order) and knowledge of COVID-19 on travel intention through attitudes is not significant (i.e., BootCI contains the number 0), but when there is a low or average level of past travel experience, the mediating effect is significant (i.e., BootCI does not contain the number 0). When there is either a low or high level of past travel experience, the mediating effect of knowledge of tourism on travel intention through attitudes is not significant (BootCI contains 0), but where there is an average level of past travel experience, the mediating effect becomes significant (BootCI does not contain 0). This suggests a lack of consistency regarding the mediating impact of attitudes across the three levels and that a conditional mediating effect exists for the three paths TI←ATT←K, TI←ATT←KT, and TI←ATT←KC. Therefore, H14a holds: past travel experience moderates the mediation of attitudes between knowledge and travel intention.

**TABLE 8:** Results of a moderated mediation analysis.

Independent variable	Mediating variable	Moderator	Effect	BootSE	BootLLCI	BootULCI
knowledge	Attitudes	Low level (−1SD)	0.042	0.024	0.001	0.091
		Mathematical mean	0.048	0.023	0.004	0.092
		High level (+1SD)	0.047	0.04	–0.026	0.130
	Subjective norms	Low level (−1SD)	0.100	0.066	–0.026	0.234
		Mathematical mean	0.182	0.042	0.102	0.267
		High level (+1SD)	0.245	0.06	0.135	0.372
	Perceived behavioral control	Low level (−1SD)	0.029	0.023	–0.009	0.079
		Mathematical mean	0.071	0.026	0.025	0.127
		High level (+1SD)	0.129	0.055	0.035	0.248
knowledge of tourism	Attitudes	Low level (−1SD)	0.024	0.015	–0.001	0.057
		Mathematical mean	0.034	0.016	0.003	0.066
		High level (+1SD)	0.036	0.032	–0.024	0.102
	Subjective norms	Low level (−1SD)	0.115	0.069	–0.013	0.265
		Mathematical mean	0.218	0.043	0.140	0.309
		High level (+1SD)	0.298	0.063	0.182	0.433
	Perceived behavioral control	Low level (−1SD)	0.031	0.023	–0.009	0.079
		Mathematical mean	0.082	0.030	0.026	0.143
		High level (+1SD)	0.153	0.065	0.031	0.288
knowledge of COVID-19	Attitudes	Low level (−1SD)	0.031	0.017	0.003	0.069
		Mathematical mean	0.035	0.015	0.006	0.066
		High level (+1SD)	0.035	0.026	–0.013	0.092
	Subjective norms	Low level (−1SD)	0.042	0.036	–0.026	0.115
		Mathematical mean	0.061	0.028	0.007	0.117
		High level (+1SD)	0.075	0.039	0.003	0.158
	Perceived behavioral control	Low level (−1SD)	0.011	0.012	–0.006	0.039
		Mathematical mean	0.023	0.012	0.004	0.052
		High level (+1SD)	0.041	0.024	0.004	0.097
perceived risk	Attitudes	Low level (−1SD)	–0.003	0.013	–0.032	0.022
		Mathematical mean	0.014	0.009	0.001	0.035
		High level (+1SD)	0.016	0.019	–0.018	0.061
	Subjective norms	Low level (−1SD)	–0.023	0.061	–0.142	0.103
		Mathematical mean	0.035	0.042	–0.047	0.121
		High level (+1SD)	0.081	0.054	–0.022	0.19
	Perceived behavioral control	Low level (−1SD)	0.019	0.029	–0.031	0.086
		Mathematical mean	0.045	0.023	0.003	0.093
		High level (+1SD)	0.072	0.038	0.005	0.152
tourism self-identity	Attitudes	Low level (−1SD)	0.013	0.022	–0.032	0.055
		Mathematical mean	0.017	0.017	–0.017	0.049
		High level (+1SD)	0.02	0.022	–0.023	0.066
	Subjective norms	Low level (−1SD)	0.222	0.080	0.081	0.400
		Mathematical mean	0.244	0.041	0.169	0.330
		High level (+1SD)	0.253	0.055	0.152	0.367
	Perceived behavioral control	Low level (−1SD)	0.028	0.023	–0.004	0.083
		Mathematical mean	0.053	0.023	0.011	0.102
		High level (+1SD)	0.085	0.043	0.008	0.176

When there is a low level of past travel experience, then the mediating effects of knowledge, knowledge of tourism, and knowledge of COVID-19 on travel intention through subjective norms and perceived behavioral control, respectively, are not significant (BootCI contains 0); however, at average or high levels of past travel experience, the mediating effect becomes significant (BootCI does not contain 0). This indicates that subjective norms and perceived behavioral control each have inconsistent mediating effects across the three levels. Therefore, for the six pathways TI←SN←K, TI←SN←KT, TI←SN←KC, TI←PBC←K, TI←PBC←KT, and TI←PBC←KC, conditional mediation exists; H14b and H14c hold.

For the mediation path TI←ATT←TID, the mediating effect of attitude is not significant regardless of the level of past travel experience (BootCI contains 0); H15a does not hold. In contrast, subjective norms do significantly mediate (BootCI does not contain 0) for the TI←SN←TID path, regardless of the level of past travel experience. At all three levels, subjective norms play a mediating role, and the size of the effect is always greater than 0. This indicates no moderation because the mediating effect is consistently the same. Thus, H15b does not hold. As for the TI←PBC←TID path, when past travel experience is at a low level, perceived behavioral control does not play a mediating role (BootCI contains 0), but it does play a mediating role at average or high levels (BootCI does not contain 0). This inconsistency in mediation across the three levels suggests conditional mediation, such that H15c holds.

For the path TI←ATT←PR, the mediating effect of attitude is not significant when past travel experience is at either a low or high level (BootCI contains 0). Here, the difference is that the effect value is negative (−0.003) at a low level and positive (0.016) at a high level. The mediating effect of attitude is, however, significant when past travel experience is at an average level (BootCI does not contain 0). Evidentially, the mediating effect of attitudes is inconsistent across the three levels; thus conditional mediation is present and H16a holds. As for TI←SN←PR, subjective norms do not play a mediating role regardless of the level of past travel experience (BootCI contains 0). There is no significant conditional mediation, and H16b does not hold. Regarding path TI←PBC←PR, perceived behavioral control does not mediate at a low level of past travel experience (BootCI contains 0) but does mediate at average or high levels (BootCI does not contain 0). This inconsistency points to perceived behavioral control’s conditional mediating role, effectively supporting H16c.

## Discussion

Perhaps no event in modern tourism has had (and continues to have) a more significant impact on travel desire, perceived travel risk, and the hospitality industry at large than the 2020 outbreak and global spread of COVID-19. As tourism destinations and management continue to grapple with the threat of outbreaks or case surges, control, and prevention measures, changing policies and various closures, the quality of knowledge on tourist subjectivities and behavior will only become more valuable. Although this study is not specifically focused on the impact of COVID-19 on risk perception (cf. Nazneen et al., 2020; Sánchez-Cañizares et al., 2021; Chi et al., 2022; Jiang et al., 2022), the pandemic is still a crucial element and inseparable background to its findings. Until the virus is defeated on the global scale, the subjective and objective effects of COVID-19 must be integrated into tourist psychology research to some degree. At the same time, it is important not to set the scope of research too narrowly and over-focus on individual reactions in the present moment, as new information is always filtered through past experiences, and potential behavior is mentally screened against the anticipated opinions and reactions of key social others. This study provides a comprehensive framework for understanding travel intention outcomes that are both adequately unique to “post-COVID” reality and sufficiently holistic for wide application.

Consistent with the conclusions of Quintal et al. (2010) and Sharifpour et al. (2014), this study finds that knowledge has a significant positive impact on travel intention. If travelers suspect that their knowledge is insufficient, they may mitigate uncertainty by abstaining from tourism activities. This, however, does not mean that the acquisition of knowledge can immediately change attitudes and affect behavioral intentions. In practice, if 40% of people exhibit a behavior, then 60% of people must have a positive attitude toward engaging in the behavior; 80% of people believe in a kind of behavior, and more than 90% must have the necessary knowledge in order for the behavior to change (Wang and Cheng, 2018). Related research has also found that more knowledge is not necessarily better, because there are negative effects associated with “information overload”. Some of the main reasons that tourists visit where they do are heterogeneity and curiosity, so when they become too familiar with a destination, curiosity weakens, as does that destination’s attractive force (Park and Jang, 2013; Hadar and Sood, 2014; Hu and Krishen, 2019). Zhu and Deng (2020) find that the greater tourists’ knowledge of tourism, the higher they will evaluate their own abilities in tourism risk management, and the stronger their travel intention will be. However, this article proposes that due to the characteristics of COVID-19—high transmissibility, a long incubation period, rapid mutation, and novelty (that is, a lack of previous experience in its prevention and control)—individual tourists will not have significantly improved perceived behavioral control with greater knowledge of COVID-19.

It is typically assumed that perceived risk undermines confidence and reduces perceived control over a situation. Conversely, this study conforms with Reisinger and Mavondo (2005); Hajibaba et al. (2017), and Vespestad et al. (2019), finding that perceived risk has a significant positive impact on travel intention. There are three possible explanations for this result: (1) Perceived risk allows individuals to understand potential threats and thus appropriately determine the level of risk, take scientific precautions, actively respond, and lessen adverse effects. (2) The situation with COVID-19-related risks is somewhat different but to a similar end. COVID-19 has yet to be completely eliminated, and threats of new outbreaks are ongoing. Even the cancellation of tourism activities may not be enough to avoid the risk of infection. Still, due to new structures or feelings of social isolation, people are eager to release psychological pressure through tourism behavior, including “getting close to nature”. Thus, the pandemic has amplified travel desires. (3) Moderate risks can actually increase the excitement of travel. In moderation, risks stimulate tourists’ adventurous spirit and drive to face challenges, which will stimulate their desire to travel as well. Some tourists will even seek out a highly volatile destination for travel (Fuchs and Reichel, 2011). The effect of perceived risk on travel intention is thus two-sided, in some contexts actually enhancing the intention to travel. This study finds that perceived risk negatively affects tourism attitudes among potential tourists, but positively affects the travel attitudes of first-time and repeat visitors. Perhaps this is because with an increase in past travel experience, tourists’ knowledge and ability to prevent and control tourism risks have been continuously enhanced, and tolerance of tourism risks has increased accordingly as well. These conclusions enrich the systematic understanding of how perceived risk affects travel attitudes and travel intention.

The study has added important concepts to the model—such as tourism self-identity and subjective norms—and put the study of individual behavioral intention into its social context, exploring how group psychological states affect an individual’s social perception, social attitude, and social behavior. Similar to Lee and Jan (2018), in this study tourism self-identity has a significant positive influence on travel intention. Compared with other influencing factors, tourism self-identity and subjective norms have a greater impact on travel intention. Therefore, tourism marketing after COVID-19 must not only focus on tourists, but also on the attitudes of potential tourists, important others, and important groups.

The study divides tourists into three categories—potential tourists, first-time visitors, and repeat visitors—to verify the moderating effect of past travel experiences. Consistent with previous findings (Sönmez and Graefe, 1998; Ajzen, 2002; Tassiello and Tillotson, 2020), when tourists have more past travel experience, there is positive moderation of the indirect effects on travel intention—through travel attitudes, subjective norms, and perceived control, perceived risk through travel attitudes, and tourism self-identity through subjective norms and perceived control. Generally speaking, the more past travel experience, the greater the effects. Similar to Ramkissoon and Mavondo (2015), which identify gender as a strong moderator of tourists’ place satisfaction and pro-environmental behavioral intention, this study’s introduction of past travel experience substantially improves explanatory power as well as the multi-dimensionality and holism of tourism behavioral modeling. Tourist psychology has much more to do with social roles and ties formed prior to or outside of the defined tourism context than is generally appreciated, and understanding the interplay of those forces requires the application of more nuanced moderated mediation models.

## Conclusion and Limitations

This study integrates TPR, TPB, SIT, and KAB models to simultaneously investigate multiple influences on travel intention, including cognitive factors and environmental factors. It builds a comprehensive model for analyzing the mechanisms by which multiple cognitive factors affect travel intention, explores the associated boundary conditions, and uses past travel experience as the moderating variable. In accounting for travel intention, the comprehensive model’s explanatory power comes to 79%, thus outperforming the TPR, TPB, SIT, and KAB models, respectively. This work verifies the combined influence of internal subjective and external objective factors on travel intention, which extends and strengthens the overall psychological-sociological framework for researching travel intention. One key finding is that subjective norms—i.e., the approval of important others—have a greater impact on travel intention than more strictly personal factors such as perceived risk and perceived behavioral control. This points to the importance of contextualizing individual tourists’ intentions within their social relations, an approach that has previously been neglected. The study also finds that, overall, knowledge can have a direct influence on travel intention as well as an indirect influence through attitudes, subjective norms, or perceived behavioral control. Identity, meanwhile, can have a direct influence on travel intention, but indirectly only works through subjective norms or perceived behavioral control, and not through attitudes. As for perceived risk, it can have a direct influence on travel intention, but only influences it indirectly through attitudes and perceived behavioral control, and not through subjective norms.

Study findings indicate that past travel experience moderates the following twelve effects: knowledge of tourism and perceived behavioral control on travel intention; knowledge, knowledge of tourism, knowledge of COVID-19 and perceived risk on attitudes; knowledge, knowledge of tourism, and identity on subjective norms; and of knowledge, knowledge of tourism, and identity on perceived behavioral control. In addition, it also positively moderates the mediating effects of attitudes, subjective norms, and perceived behavioral control. The richer a tourist’s past travel experience, the greater the effect (repeat visitors > first-time visitors > potential tourists). In that sense, this study tests the influence of practical knowledge on tourists’ perceptions and travel decisions. Compared with indirect experience (knowledge of tourism and knowledge of COVID-19), the direct, practical experience of past travel has a greater impact on tourists’ perceptions and travel decisions. This shows that the source of knowledge is important, leading to different cognitive and behavioral outcomes. Therefore, research on the relationship between tourists’ cognitive factors and tourism attitudes or behavioral decisions will benefit greatly from the introduction of the independent variable “past travel experience.”

This study’s comprehensive model lays the groundwork for better decision-making in tourism management. Finding that subjective norms are the most important factor influencing travel intention, implies that post-COVID tourism marketing must focus not only on the tourists themselves but on the attitudes of potential tourists, important others, and important groups as well. Analysis of subjective norms’ antecedent variables finds that knowledge and self-identity significantly improve tourists’ evaluation of important others’ travel behavior approval, whereas perceived risk does not lead to an improvement in subjective norms. Therefore, tourism destinations can improve tourists’ evaluation of subjective norms by various routes including promoting knowledge of tourism risks and enhancing tourism self-identity. At the same time, these methods can improve tourists’ sense of perceived behavioral control, which in turn reinforces travel intention. Because perceived risk positively affects travel intention, the development of abundant and stimulating experiential tourism products is crucial to improving travel intention. In comparing the specific dimensions of perceived risk, this study finds that potential tourists are most concerned about cost risks; therefore, tourist receiving locations should actively mitigate the risks of economic and time costs that tourists face—by setting reasonable prices, by better-publicizing traffic, tour route and tourism product information and by improving the quality of tourism services. Moreover, in light of the current epidemic situation and tourists’ concomitant psychological fluctuations, destinations must implement normalized epidemic prevention measures to create a safe and comfortable tourism environment.

This study does have its limitations. First, although the predictive power of the comprehensive model exceeds that of competing models, it still falls short of completely explaining travel intention, indicating that there are other factors at play. This study only selected typical cognitive factors to verify their influence on travel intention. In reality, tourists’ behavioral decisions may also be affected by emotional factors (e.g., worry) and other cognitive factors (perceived value, satisfaction, etc.). Follow-up studies may construct a more complete theoretical model. Second, the study finds that knowledge has a significant positive impact on travel intention, but it is known that information overload reduces the attractiveness of destinations to tourists, in turn reducing their travel intention (Hadar and Sood, 2014; Hu and Krishen, 2019). Resolution of this apparent paradox will require follow-up studies to compare the effect boundaries for different levels of knowledge affecting behavioral intentions in greater detail, thereby identifying the thresholds at which knowledge changes from a positive to a negative factor for travel intention.

## Data Availability Statement

The original contributions presented in this study are included in the article/supplementary material, further inquiries can be directed to the corresponding authors.

## Ethics Statement

The studies involving human participants were reviewed and approved by College of Economics, Southwest Minzu University. Written informed consent to participate in this study was provided by the participants’ legal guardian/next of kin.

## Author Contributions

XJ conceived and designed the concept, collected the data, and wrote the manuscript. JQ provided technical support and supervision. JG was responsible for investigation, data collection, and validation. MG reviewed and edited the manuscript. All authors have read and agreed to the published version of the manuscript.

## Conflict of Interest

The authors declare that the research was conducted in the absence of any commercial or financial relationships that could be construed as a potential conflict of interest.

## Publisher’s Note

All claims expressed in this article are solely those of the authors and do not necessarily represent those of their affiliated organizations, or those of the publisher, the editors and the reviewers. Any product that may be evaluated in this article, or claim that may be made by its manufacturer, is not guaranteed or endorsed by the publisher.

## Footnotes

1
https://www.wjx.cn/
